# Toward practical issues: Identification and mitigation of the impurity effect in glyme solvents on the reversibility of Mg plating/stripping in Mg batteries

**DOI:** 10.3389/fchem.2022.966332

**Published:** 2022-08-12

**Authors:** Zhenzhen Yang, Mengxi Yang, Nathan T. Hahn, Justin Connell, Ira Bloom, Chen Liao, Brian J. Ingram, Lynn Trahey

**Affiliations:** ^1^ Chemical Sciences and Engineering Division, Argonne National Laboratory, Lemont, IL, United States; ^2^ Joint Center for Energy Storage Research, Argonne National Laboratory, Lemont, IL, United States; ^3^ Material, Physical, and Chemical Sciences Center, Sandia National Laboratories, Albuquerque, NM, United States; ^4^ Materials Science Division, Argonne National Laboratory, Lemont, IL, United States

**Keywords:** reversibility, Mg plating/stripping, glyme solvent, Mg (TFSI)_2_, impurity effect

## Abstract

Reversible electrochemical magnesium plating/stripping processes are important for the development of high-energy-density Mg batteries based on Mg anodes. Ether glyme solutions such as monoglyme (G1), diglyme (G2), and triglyme (G3) with the MgTFSI_2_ salt are one of the conventional and commonly used electrolytes that can obtain the reversible behavior of Mg electrodes. However, the electrolyte cathodic efficiency is argued to be limited due to the enormous parasitic reductive decomposition and passivation, which is governed by impurities. In this work, a systematic identification of the impurities in these systems and their effect on the Mg deposition–dissolution processes is reported. The mitigation methods generally used for eliminating impurities are evaluated, and their beneficial effects on the improved reactivity are also discussed. By comparing the performances, we proposed a necessary conditioning protocol that can be easy to handle and much safer toward the practical application of MgTFSI_2_/glyme electrolytes containing impurities.

## 1 Introduction

Multivalent energy storage technologies based on Mg, Zn, or Ca are attracting increasing attention due to their high volumetric capacities (i.e., 3,832 mA h/cm^3^ Mg vs. 2,062 mA h/cm^3^ Li and 1,136 mAh/cm^3^ Na) and low cost due to their natural abundancy (Yoo et al., 2013; Muldoon et al., 2014). Significant efforts have been devoted in the search for an optimal combination of electrodes/electrolyte materials since Aurbach reported the first rechargeable Mg battery prototype (Aurbach et al., 2000; Aurbach et al., 2007). However, the development of useful electrolytes, exhibiting a wide electrochemical window with suitable compatibility with anode and cathode materials, is still very limited. Years of fundamental studies have shown that the electrolyte properties such as conductivity, viscosity, solvation structure, and chemical stability greatly affect the electrochemical performance (Deivanayagam et al., 2019; Leon et al., 2022) and are highly dependent on the exact formulation and a complex interaction between solvents and salts. With these constraints, only a few solvents meeting the requirements are currently practical in terms of their ability to dissociate Mg (or Zn and Ca) salts and show reversible metal plating and stripping processes (Bitenc et al., 2019; Deivanayagam et al., 2019). Among them, ethereal solvents such as glymes are perhaps most commonly employed due to low viscosity, high chemical stability, and relatively low vapor pressure for multivalent systems. For example, magnesium bis-(trifluoromethanesulfonyl) imide [Mg (TFSI)_2_] salt (Shterenberg et al., 2015) or magnesium carba-closo-dodecaborate [Mg (HCB_11_H_11_)_2_] in glyme solvents has been intensively explored as promising candidates for Mg batteries (McArthur et al., 2017; Hahn et al., 2018; Fisher et al., 2019).

Despite these attractive characteristics, these electrolytes exhibited poor Coulombic efficiency for Mg deposition and stripping (Connell et al., 2016; Sa et al., 2016). The origin of their poor electrochemical performance is still debated due to conflicting experimental observations and theoretical explanations of the Mg anode passivation mechanisms. Recent studies have suggested that the presence of trace levels of chemical impurities from solvents and anions can affect the material properties substantially (Connell et al., 2016; Kang et al., 2019). Even at very low concentrations, the impurities in the glyme system play an important role in determining the degree of reversibility of magnesium deposition/stripping. For example, it was reported that trace levels of H_2_O (≤ 3 ppm) have a profound impact on the reversibility of Mg deposition and stability at the electrode/electrolyte interface, as well as passivation behaviors (Connell et al., 2016). However, detailed investigations of other chemical impurities in addition to water have not been reported yet. Generally, impurities in solvents used for formulation can come from synthesis/manufacturing processes, degradation, storage conditions, or chance contamination. Similarly, impurities from raw materials can react with various chemicals or the atmosphere to form other substances, like reactive intermediates or degradation products during electrolyte preparation and storage, which have the potential to affect the performance as well.

Currently, MgTFSI_2_ is one of the few simple salts known that can be dissolved in many organic solvents and show high anodic stability, so it is the most commonly used ether-soluble salt for Mg batteries (Ha et al., 2014; Shterenberg et al., 2015). Herein, as a benchmarking system and commercially available material, we specifically focus on the details of MgTFSI_2_ in glycol dimethyl ether solutions (Gx) including monoglyme (G1), diglyme (G2), and triglyme (G3). We systematically identified the impurities present in these systems and their effect on electrochemical performance. Mitigation strategies to remove the impurities and improve the activity are assessed, including ordinary purification methods, adding electrolyte additives, and electrochemical conditioning. Each mitigation method is investigated to elucidate why performance is amended. Finally, we propose a facile conditioning process based on electrochemical galvanostatic cycling, which is the necessity step in practical use to maximize the Coulombic efficiency of Mg deposition–dissolution. We believe that the impurity issues are not limited to MgTFSI_2_/Gx but are generic to most electrolyte systems for the Mg battery, so this work will provide an effective approach to optimize and achieve the advanced performance for more practical electrolyte systems.

## 2 Results and discussion

### 2.1 Identification of impurities and performance with as-received Gx solvents

**FIGURE 1:**
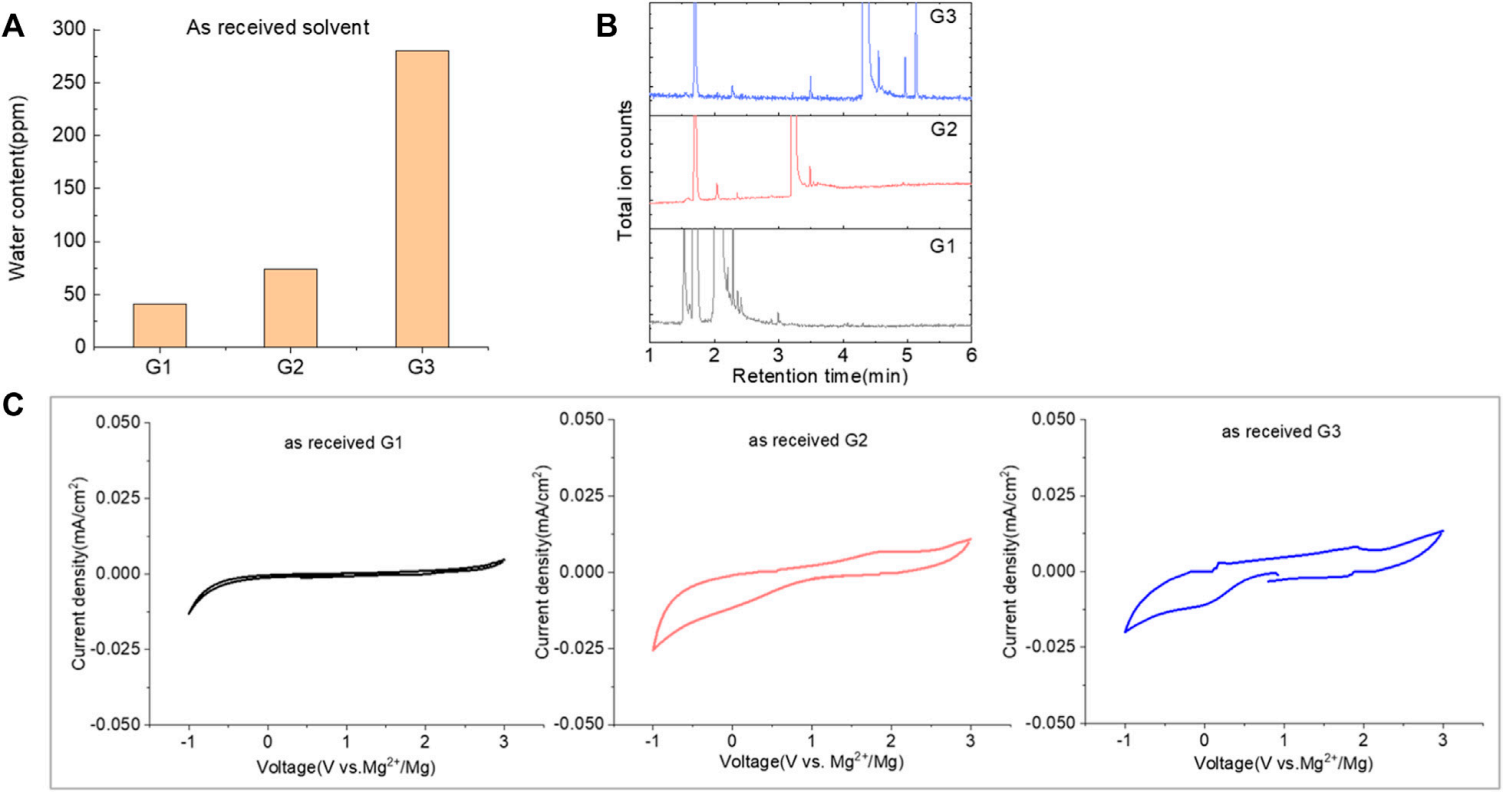
**(A)** Water content in the as-received solvents from G1 to G3 by Karl Fischer titration. **(B)** Chromatograms (total ion count) of the as-received solvents in G1, G2, and G3 by GC-MS. **(C)** Cyclic voltammogram of the freshly prepared electrolyte using “as-received” G1, G2, and G3 with 0.5 M MgTFIS_2_ on a Pt electrode. Scan rate: 25 mV/s.

Glymes usually are synthesized by several common methods at large scales using ethylene epoxide and alcohols with Lewis acid catalyzed at high temperature and pressure (Tang and Zhao, 2014). The common materials and routes to prepare glymes are shown in Supplementary Figure S1. The most common impurity expected in the “as-received” Gx is small amounts of water. Most glymes are completely miscible with water and alcohols so that its presence is inevitable. Therefore, the Karl Fischer titration was employed to determine the water content in the “as-received” Gx solvent, and the results are given in Figure 1A. It is clear that the water content increases from G1 to G3 as the chain increases in the glyme chemistry, which is indicative of the added chemical steps or larger alcohols needed to go from one to another. Moreover, the polarity including dipole moments and dielectric constants increases with the ethylene oxide chain length (Tang and Zhao, 2014); thus, higher polarity may also contribute to the higher measures of water content from G1 to G3.

Because of their manufacturing process, some other organic impurities such as diethyl ether and hydrocarbons could be found in glymes as well. Gas chromatography-mass spectrometry (GC-MS) is ideally suited for the determination of the trace organic impurities due to its excellent sensitivity. The total ion current chromatograms (TICs) obtained from GC for the “as-received” Gx are given in Figure 1B. Due to trace amounts of impurities, the chromatograms were magnified to reveal the small impurity peaks in glymes (the large peaks are due to the solvent itself). It shows the raw material from the supplier contained impurity peaks primarily located in the 1–6 min region. The peaks were assigned to the most probable candidates from the NIST library, as provided in Table 1. We hypothesize that these impurities could be the residues of unconverted starting reactants such as propylene oxide and alcohols, or from the intermediates such as 2-ethoxyethanol (see Supplementary Figure S1), or the result of the instability of glymes at high temperatures possibly arising in an industrial plant during production, purification, and even packing. The increase of impurities with the chain length can probably be explained by the increasing boiling point with the chain length of the glymes, which complicates their purification as it requires higher temperatures in the purification process so that additional decomposition processes may occur. As a result, any such common products that may be produced in the synthesis would be hard to monitor in the commercial solvents. In fact, it is difficult to remove all the impurity substances effectively and fully at a large scale by manufacturing processes unless proper care is taken in every step involved with the increased cost.

**TABLE 1:** Impurity identification in the as-received solvents by GC-MS.

Solvent	RT (min)	Possible compound
G1 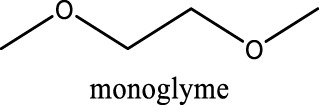	1.70	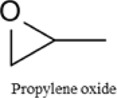
	2.29	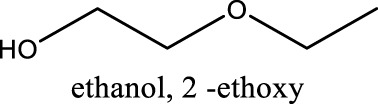
	2.42	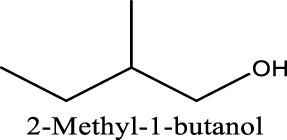
	2.99	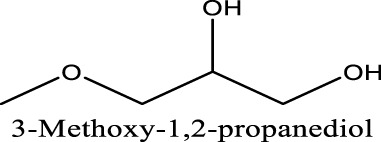
G2 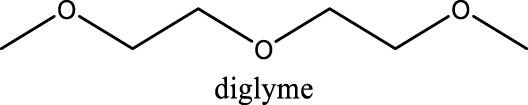	1.70	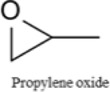
	2.04	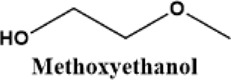
	2.36	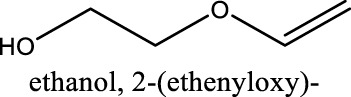
	3.48	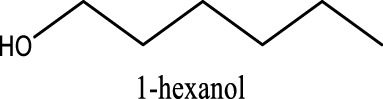
	3.55	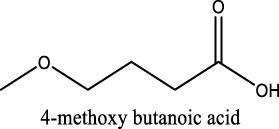
G3 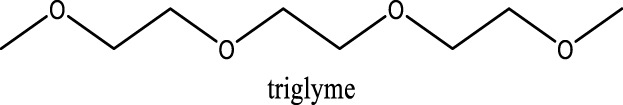	1.70	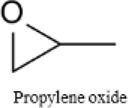
	2.3	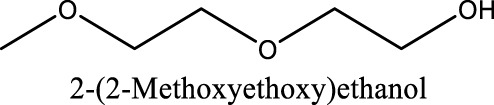
	3.38	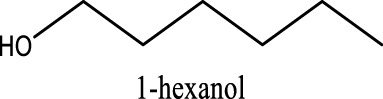
	4.57	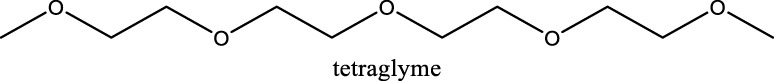
	4.97	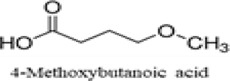
	5.15	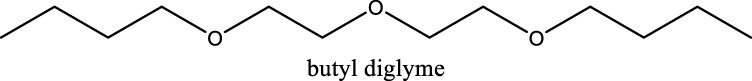

Figure 1C shows the first CV result from the freshly prepared solution based on the “as-received” G1, G2, and G3 containing 0.5 M MgTFSI_2_ using a Pt working electrode. A reductive process occurs at an onset higher than 0 V, and no oxidative current is obtained in positive scan in the CV curve. This small reductive current is attributed to irreversible processes such as the formation of a passivation film on the electrode surface, rather than Mg deposition. Not surprisingly, the “as-received” solvent cannot support any reversible Mg deposition/stripping, regardless of the solvent used.

### 2.2 Improved performance: Solvent purification processing

**FIGURE 2:**
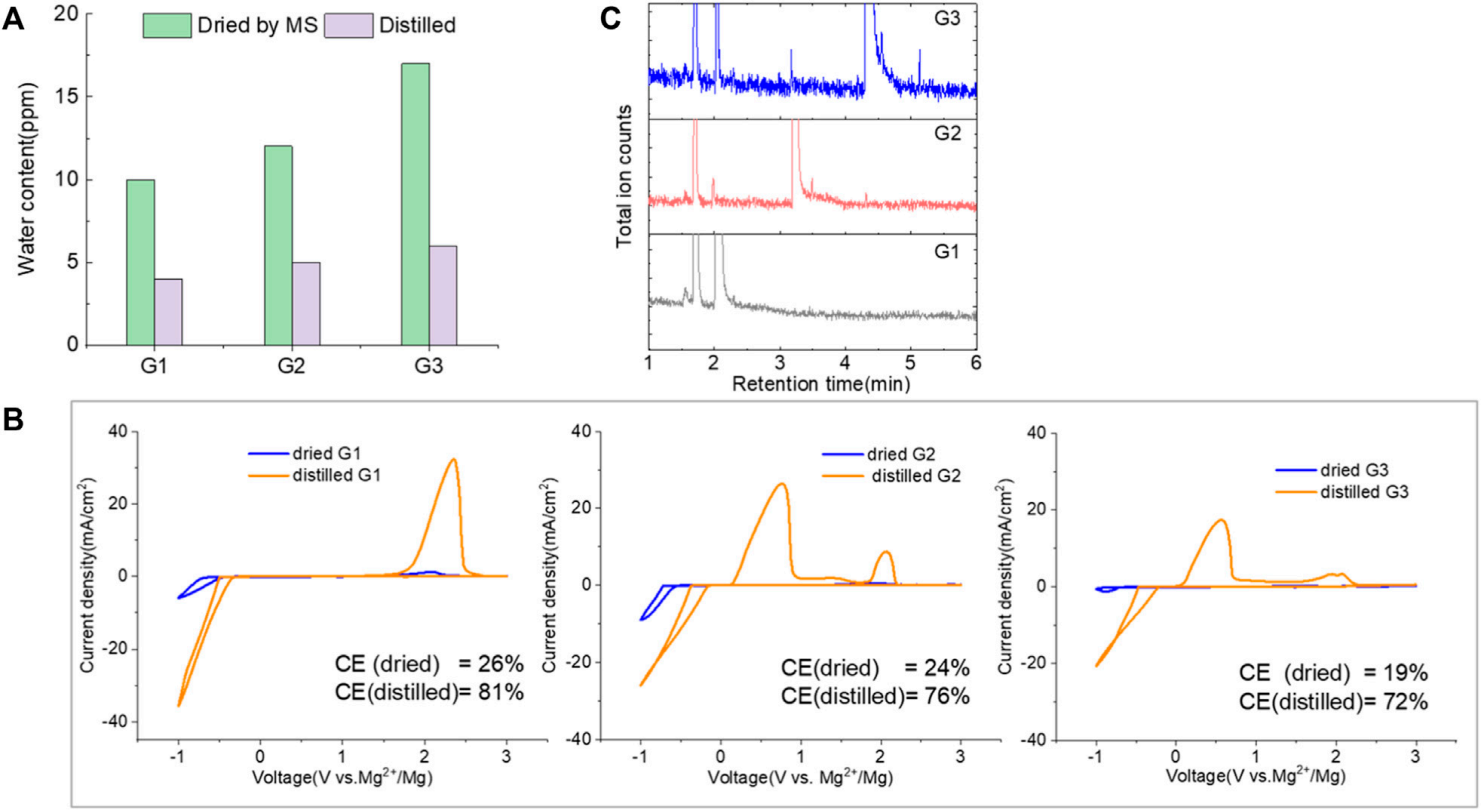
**(A)** Water content in the solvents dried by molecular sieve, distilled over Na/K measured *via* Karl Fischer titration. **(B)** Cyclic voltammograms (CVs) for the electrolytes with 0.5 M MgTFSI_2_ in pretreated G1, G2, and G3 dried by molecular sieves and distillation methods. Pt was used as the working electrode and a scan rate of 25 mV s^−1^. **(C)** Chromatograms (total ion count) of the distilled solvents in G1, G2, and G3 by GC-MS.

The purity of glymes is confirmed to be critical to enable the efficient Mg electrochemical activity. Typical purification treatments to remove impurities from hydrophilic solvents are adsorption with molecular sieves (MS) and distillation. As noted in Figure 2A, the storage of the “wet” glymes over 3-Å molecular sieves for 48 h readily provided “dry” solvent with moisture content in the 10–20 ppm range. Distilling over Na/K can reduce the water content < 10 ppm. We evaluated the relative effects of absorption and distillation purification methods on Mg deposition/stripping in MgTFSI_2_-Gx solutions, as shown in Figure 2B. Compared to the electrochemical activity from “as-received” Gx solvents, both methods show evidence of increased Columbic efficiency (CE) and current density upon Mg deposition and dissolution; however, the distillation purification approach results in significantly enhanced performance. This suggests a “relatively clean” solvent was obtained by distillation and confirms that water impurity plays a critical role in controlling the nature of Mg deposition/stripping, consistent with previous reporting (Connell et al., 2016).

Also it should be noted that regardless of the solvent, the onset plating voltage in the distilled solution tends to be more positive (−0.2 V vs. Mg^2+^/Mg) compared to that in the dried solution (−0.5 V vs. Mg^2+^/Mg), indicating that the beginning of plating requires a higher overpotential activation for the dried solution due to more passivation at the electrode interface from impurities. This general phenomenon does not occur on the opposite polarization. The subsequent Mg stripping voltage starts from ∼2.0 V for all the dried solutions. However, a single oxidation peak appears at 2.0 V for distilled G1, and a large oxidation peak occurs at ∼0.4 V with a smaller peak near 2.0 V for distilled G2 and G3. This overpotential decrease probably suggests that the reaction products formed from the side reaction between Mg^0^ and the electrolyte components (e.g., SEI) make the dissolution of Mg^0^ easier in distilled G2 and G3 than that in distilled G1. The difference in the interfacial properties with different solvents inspired us to further explore and understand the fundamental mechanism for these behavior changes and will be the subject of a separate report.

We noted that a trend was found using both approaches that the performance decreases in the order of G1>G2>G3. The preferred solvation structures formed in G1–G3 affect the performance, which has been discussed in the literature (Seguin et al., 2019). Although with pretreatments, more impurities still existing in the glymes with longer chains are also partially responsible for the performance difference. Despite containing similar water contents, the distinct difference in the performance between distilled G2 and G3 suggests that other organic impurities contribute to CE. The GC-MS measurement in Figure 2C reveals that the signals of some small alcohol impurities such as 2-ethoxyethanol and 1-butanol are present in the as-received G1 solvent and disappear after distillation; however, larger alcohols are not completely removed by distillation in G2 and G3. The role of organic impurities on the performance is discussed in Section 2.4.

By evaluating the performance, we demonstrated that most of the moisture along with some small organic impurities can be successfully removed from “as-received” solvents by the Na/K distillation process. Therefore, this method is employed to ensure solvent purity and provide better performance at the laboratory scale. Nevertheless, there is a risk associated with this aggressive purification method utilizing reactive Na/K of personal injury and incurred liability (Michaels, 2000). To mitigate this risk, alternative purification approaches including the use of molecular sieves and calcium hydride (CaH_2_), which are convenient and often the drying agent of choices in the purification of laboratory solvents, can rapidly provide relatively dry glymes suitable for the Mg battery. Mg deposition/stripping efficiency (data for CaH_2_ drying are shown in Supplementary Figure S2), however, is lower than that from Na/K distillation (∼20% vs. ∼70%), suggesting that the drying agent composition plays a role in removing impurities.

### 2.3 Improved performance: Incorporating supporting additives and co-salt

**FIGURE 3:**
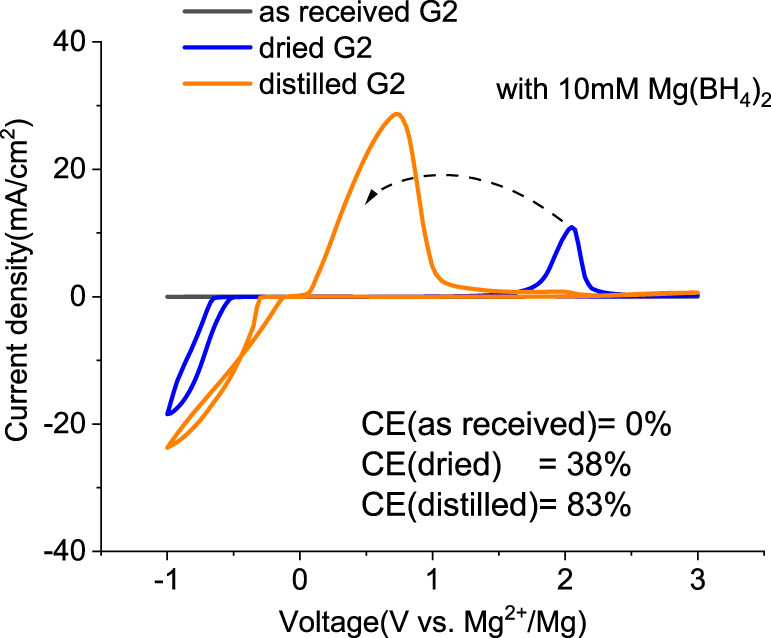
First cyclic voltammograms (CVs) for the electrolytes with 0.5 M MgTFIS_2_ and 10 mM Mg (BH_4_)_2_ in G2 from as-received solvents, dried by molecular sieves, and distilled. Pt was used as the working electrode, and the scan rate was 25 mV s^−1^.

Mitigating the impurity issues and enhancing performance can be achieved through alternative paths. Adding reductive scavengers, such as Mg metal powders, Bu_2_Mg, CrCl_3_, and AlCl_3_ in THF-based solutions, is an effective way to remove the interfering impurities, yet these methods rely on the use of flammable Mg powder or toxic CrCl_3_, which are not practical for commercial manufacturing (Ha et al., 2016; Deivanayagam et al., 2019; He et al., 2019). Recently, Mg(BH_4_)_2_ has been proposed as an effective water scavenging species in Mg (TFSI)_2_/tetraglyme electrolyte (Ma et al., 2017). Thus, MgTFSI_2_/G2 electrolytes were prepared with 10 mM Mg(BH_4_)_2_. The resulting CVs are presented in Figure 3. For as-received G2, the CV matches the data in Figure 1C, and no improvement is evident after Mg(BH_4_)_2_ addition. The performance in dried solvent (MS) exhibits reversible behavior with improved CE from 24% to 38%. The CE of distilled G2 systems is enhanced from 76% to 83% upon Mg (BH_4_)_2_ addition. The trend indicates that supporting additives like Mg(BH_4_)_2_ are optimized in extrinsically dried or distilled solvents to minimize the water content. The exact water content cannot be easily determined in the solutions by the Karl Fischer analysis due to the interference from the residual BH_4_^−^ (Ma et al., 2017). More recently, Wang et al. (2020) have reported that adding 0.1 M Mg (BH_4_)_2_ improves the performance in MgTFSI_2_/G2 and revealed that the mechanism was due to the preferred adsorption of BH_4_^−^ anions on the Mg metal surface. But considering the much higher amount they used, it would be the same strategy as we discussed as follows as a co-salt system rather than as the supporting additive.

**FIGURE 4:**
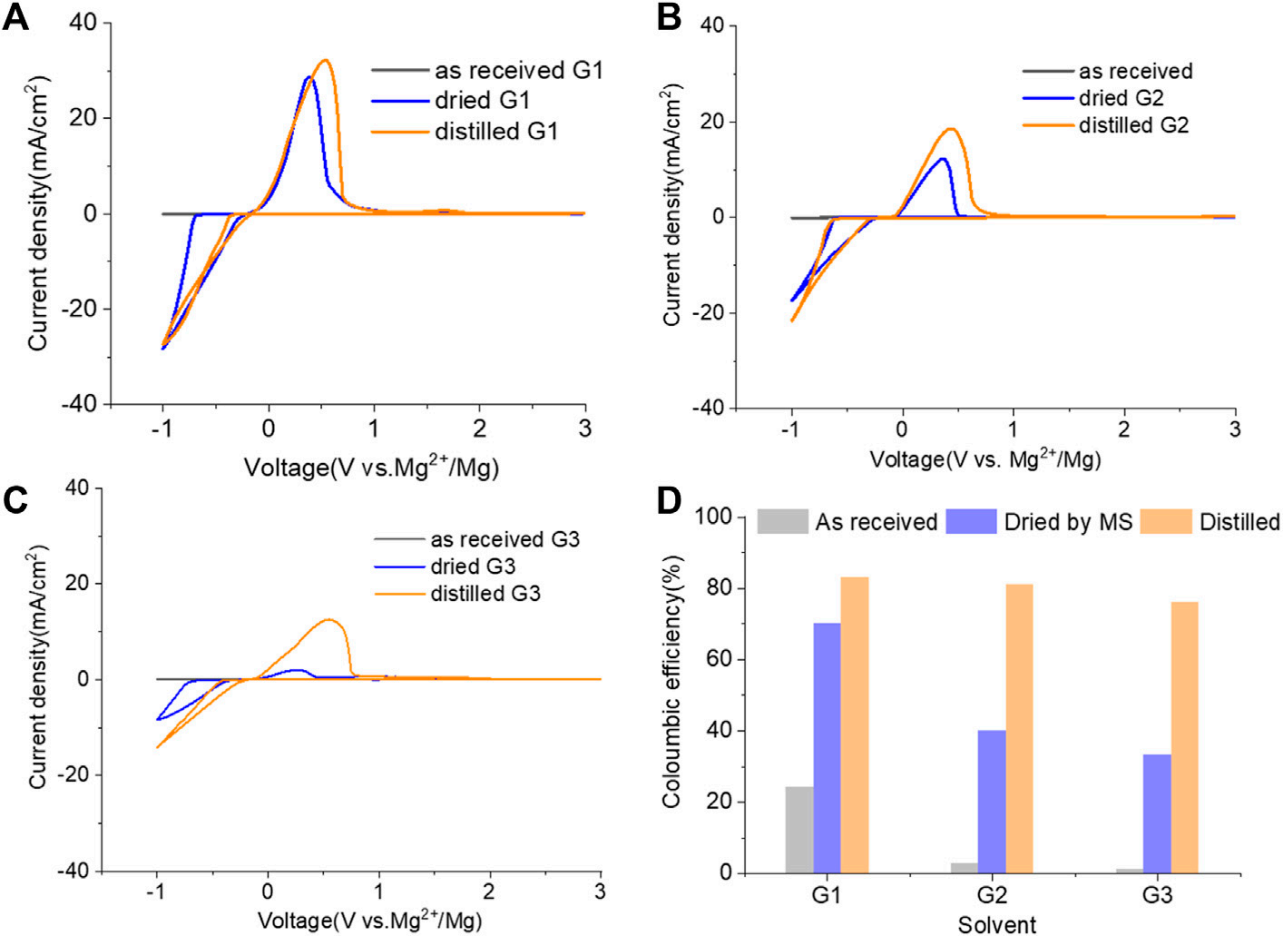
Cyclic voltammograms (CVs) for the electrolytes in **(A)** G1, **(B)** G2, **(C)** G3, and **(D)** Coulombic efficiency for each solvent (dried by molecular sieves, distilled) containing 0.25 M MgTFIS_2_ and 0.25 M MgCl_2_. Pt was used as the working electrode, and the scan rate was 25 mV s^−1^.

Inspired by the electrolyte composition and a better reversibility in the magnesium–aluminum–chloride complex (MACC), the reintroduction of chlorides, such as MgCl_2_, is another successful method to boost the MgTFSI_2_/Gx performance and is widely employed by the community (Barile et al., 2014; Pan et al., 2016; Sa et al., 2016). However, these “additions” are far from being similar to the addition of a small amount of supporting additives adopted earlier because it requires a substantial quantity of MgCl_2_ to combat the negative effects of impurities and support magnesium deposition at high efficiency (Connell et al., 2016; Connell et al., 2020). In this work, 0.25 M MgCl_2_ was added to 0.25 M MgTFSI_2_/Gx (1:1 ratio for Cl:TFSI), resulting in a total Mg^2+^ concentration of 0.5 M to compare the MgCl_2_ effect on the electrolyte performance. Figure 4 (A–C) displays the results of the first CV, a Pt working electrode in solutions of “as-received,” “dried,” and “distilled” Gx based on 0.25 M MgCl_2_ and 0.25 M MgTFSI_2_. As expected, adding MgCl_2_ to the MgTFSI_2_/Gx solutions increases the overall electrochemical properties in terms of current density and CE (Figures 4D vs. Figures 1C and 2B) for all solvents with different treatments. Compared to the results in Figure 2C, the onset potential for the stripping peak is also greatly reduced with the addition of MgCl_2_. For example, in G1 solution, it shifts from ∼2V to 0.5 V during positive scanning, implying less passivated Mg formed in the cathodic scan. With the presence of more impurity residues in the “as-received,” however, the improvement in CE was limited even with the addition of MgCl_2_. The mixed co-salt MgCl_2_/MgTFSI_2_ in dried and distilled solvents restores the CE. Again, the results suggest any beneficial effect that Cl^−^ has can be maximized in the “clean” electrolyte with much lower concentration of impurity residues.

**FIGURE 5:**
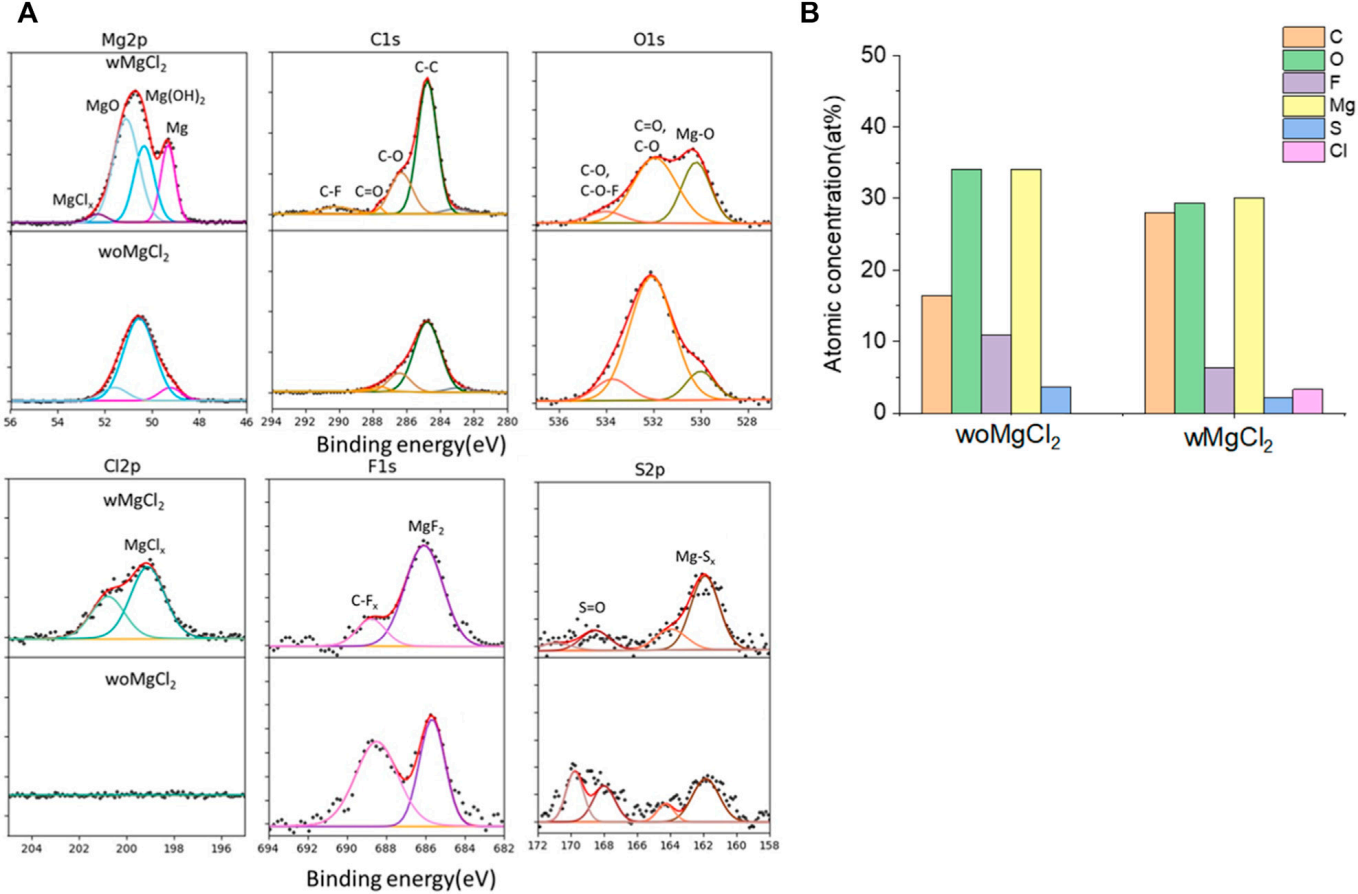
**(A)** High-resolution XPS spectra of Mg 2p, C1s, O1s, Cl 2p, F 1s, and S 2p on the surface layer of the cycled Mg electrode with/without MgCl_2_ in MgTFSI_2_/G2 dried solutions. **(B)** Atomic percentage (at%) of the SEI on Mg electrodes cycled with/without 0.25 M MgCl_2_ in MgTFSI_2_-G2 electrolytes.

The twofold advantages of MgCl_2_ are responsible for the enhanced reversible Mg cycling behavior: the dramatic changes in the solvation structure in the bulk electrolyte (i.e., formation of Mg_x_Cl_y_ complex cationic species and an interplay between TFSI and Cl coordination) and interface speciation upon the addition of MgCl_2_ to the electrolyte (Connell et al., 2016). The changes in bulk electrolyte characteristics with MgCl_2_ including ionic conductivity, ionic speciation, and anion association strength are reported elsewhere and not discussed in this work (Sa et al., 2016; Salama et al., 2017; Connell et al., 2020). Herein, we investigated the influence of MgCl_2_ on surface chemistry because the interface formation on the Mg electrode is highly susceptible to impurities and can be significantly changed by Cl^−^. To verify the beneficial role of MgCl_2_ on the formation of the surface layer, Mg electrodes cycled in dried MgTFSI_2_/G2 with and without MgCl_2_ electrolytes are characterized by XPS. Figure 5A displays the high-resolution spectra of Mg2p, Cl2p, S2p, C1s, O1s, and F1s on the Mg surface with the detailed analysis of the composition of the surface layer by carefully fitting the XPS spectra. It is immediately evident that with MgCl_2_, the electrode has a smaller amount of MgO/Mg (OH)_2_, resulting in more accessibility of the underlying active metallic Mg, estimated by a much higher Mg^0^ peak at 49.4 eV in Mg2p spectrum. The active species of Mg_x_Cl_y_^−^ cation in addition to TFSI-coordinated Mg^2+^ in the solution can precede the electron transfer and be involved on the interface, causing Mg–Cl bonding to be present in the Cl2p region around 200 eV on the surface layer. Similar results using Pt as a working electrode have been reported in the previous work (Connell et al., 2016). The presence of O = SO, Mg_x_S in the S2p region, and −CF_x_, MgF_2_ peaks in the F 1s region suggest the decomposition of TFSI^−^ in both electrolytes. The instability of TFSI^−^ and its reduction on the Mg anode are also observed in the following section after extensive cycling (i.e., “conditioning”). The carbon spectra in each electrolyte feature a major peak at 286 eV and a smaller peak at 288 eV, which is the characteristic of C–O–C and O–C = O, respectively, attributed to the decomposition of G2. However, the atomic percentage of surface composition (at% summarized in Figure 5B) is dominated by higher carbon and a lower content of F with MgCl_2_. It appears that TFSI decomposition is less extensive but rather solvent decomposition is more in the case of mixed MgCl_2_/MgTFSI_2_-electrolyte than the pure TFSI solution. It reveals that chloride addition affects the reaction of both the TFSI anion and the solvent during surface layer formation. Although with a certain level of impurities in the dried electrolyte, Cl^−^ can competitively react/more strongly bind to the Mg surface and depress the overall coverage by MgO/Mg (OH)_2_ from impurities, as well as inhibit TFSI from decomposition to some extent.

### 2.4 Improved performance: Electrolytic conditioning

It was shown that even rigorously cleaned MgTFSI_2_/glyme solutions are limited to a Coulombic efficiency of ∼70% for Mg deposition/dissolution during the first cycle. The strategies with adding reducing agents are significant advancements toward the implementation of the MgTFSI_2_/Gx electrolyte, but the practical CE values have not been obtained. To further improve the performance, earlier reports focused on electrochemical conditioning by extensive repeated CV cycling between −1.2 and 2.8 V at a slow scan rate for 200–500 cycles (Barile et al., 2014). Nevertheless, to the community, the term “conditioning” does not have a formal definition, and in many cases, it simply implies “cycling until it works better.” Consequently, the disadvantage of the traditional conditioning methods using the potential-controlled cycling is the lack of quantifiable evaluation for optimized performance. Hereafter, we proposed a facile conditioning protocol for practical applications, modified based on the galvanostatic cycling suggested by a previous report (Shterenberg et al., 2015). Our conditioning method within an electrochemical cell (Figure 9 shown in experimental) entails attaching the WE lead to one Mg rod; the CE/RE leads to the second Mg rod and cycling Mg^2+^ back and forth between the rods at a fixed current while changing the direction of flow every 1,000 s. Typical current levels in our cells are 0.1–1 mA depending on the electrolyte status and electrode surface area. The overall charge per volume required to attain the optimized electrochemical response can be as little as 5 C/ml (1 C = 1 mA × 1,000 s) or as much as 20–30 C/ml, depending on the electrolyte purity, shelf life, etc. The CV response on a third Pt electrode is verified periodically (i.e., every 5–10 C/ml) to assess the progress. Supplementary Figure S3 illustrates the two-electrode electrolysis conditioning process using “as-received” G2 as an example. A large current of 1 mA was used in the first step to start conditioning for 10 ks, as shown in Supplementary Figure S3A. CV measurement was carried out, and CE was calculated to check if there is any improvement, as shown in Supplementary Figure S3B. The cell polarization decreases gradually, indicated by voltage vs. time profiles in Supplementary Figure S3A, demonstrating that conditioning is still working. After 1 mA conditioning, a slower conditioning process was applied using lower current, e.g., 0.5 mA or less, as the electrolyte becomes cleaner. In theory, as the impurities become more dilute and the flux of impurities to the surface is decreased, they cannot diffuse toward the Mg surfaces as quickly, so a lower current/longer time conditioning protocol might be needed toward the end. Supplementary Figure S3B shows the changes in the CV measurement of the electrolyte throughout a typical conditioning process. A reductive feature (∼−0.5 V) ascribed to Mg deposition and a corresponding small stripping peak (0.5 V) appear after passing 10 C/ml in the first conditioning step. This deposition and stripping feature evolve in character in the following condition cycles using lower currents. The current density increases significantly, and CE also improves from 25% to 81% for the “as-received” solution after passing 30 C/ml in the electrolyte. The conditioning process is stopped at this point because we found that the CE cannot be improved any further as conditioning continues. CE in fact decreases if overdose of conditioning is applied.

**FIGURE 6:**
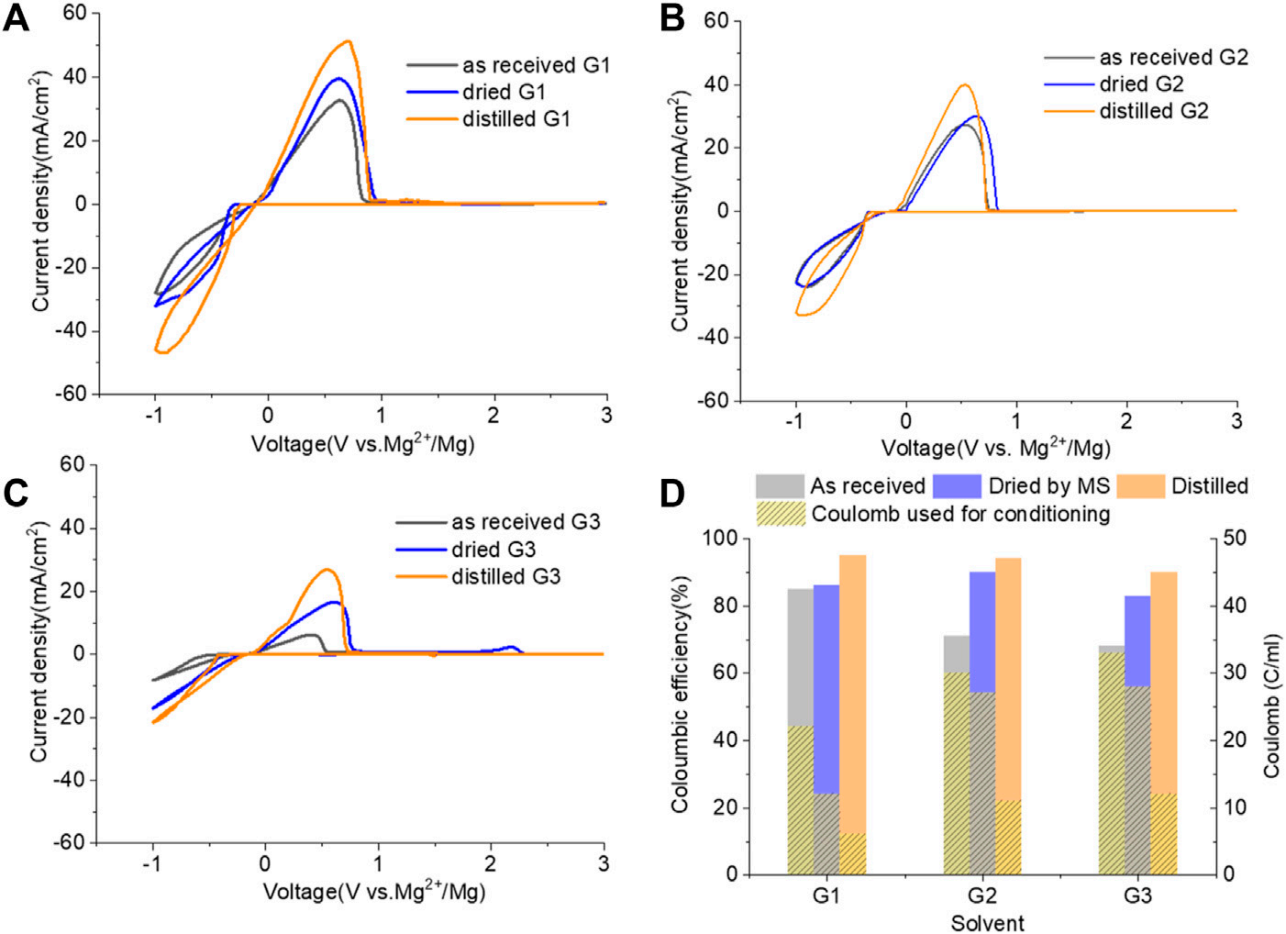
Cyclic voltammograms (CVs) for the electrolytes in **(A)** G,1 **(B)** G2, and **(C)** G3 (as received, dried by molecular sieves, and distilled) containing 0.25 M MgTFIS_2_ and 0.25 M MgCl_2_ after conditioning. Pt was used as the working electrode, and the scan rate was 25 mV s^−1^. **(D)** Columbic efficiency from CV in each used solvent after conditioning. The filled pattern indicates the total charge used for conditioning to eliminate impurities and optimize the reversibility of Mg deposition/stripping.

Figure 6 demonstrates the CVs of Mg plating/stripping in the solutions, which contain “as-received,” “dried,” and “distilled” G1, G2, and G3 solvents using the same electrolytes from Figure 4 after the conditioning process. CE of each testing solution and the total charge used for conditioning to eliminate impurity residues and optimize the efficiency are provided in Figure 6D. After conditioning, the CE (and the current density) improves but decreases in the order of G1 > G2 > G3. On the other hand, the conditioning cycles and total charge used for conditioning particularly in “as-received” and “dried” solutions increase in the order of G1< G2 < G3. Lower CE is obtained in G2 and G3 even using the similar amount of charge during the conditioning process. This again implies that more impurities are present in the longer chain Gx.

It is critical to initiate testing with highly pure materials to minimize the conditioning requirements. We found that in most cases for MgTFSI_2_/Gx systems, conditioning is required, nonetheless, to maximize Mg deposition/stripping efficiency and obtain relatively constant electrochemical results. One possibility for the observed low CE is the ubiquity, solubility, and proclivity of H_2_O to physisorb on reaction container surfaces. Although the “conditioning-free” electrolytes with additives such as heptamethyldisilazane (HpMS) can scavenge water, the CE was still below 70% due to trace water left (Kang et al., 2019). As such, achieving anhydrous conditions is always questionable and challenging. The fundamental question “How dry is dry?” proves to be difficult to answer if an accurate determination of a trace water contaminant lower than the ppm level (<3 ppm) is required.

**FIGURE 7:**
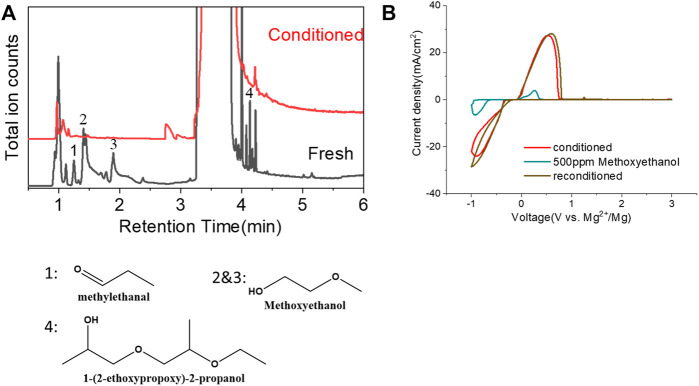
**(A)** Chromatograms (total ion count) of the distilled solvents G2 for the fresh electrolyte and conditioned electrolyte by GC-MS. **(B)** CV measurement before and after intentionally adding 500 ppm methoxyethanol in the conditioned solution. The performance is restored to the conditioned values after performing conditioning again. Scan rate: 25 mV/s.

Another possibility is some protic organic contaminants (R-OH) such as ethyl glycol, as discussed in Section 2.2, are not readily removed, even by rigorous distillation processes. Electrolytic conditioning can break down these protic residual molecules, evidenced from GC-MS data before and after the conditioning process in distilled MgTFSI_2_/G2 solutions as a case study, as shown in Figure 7A. We observed that the signals for longer alcohols such as methoxyethanol and propanol disappear after conditioning. To confirm the inhibitory effect of organic impurities, we intentionally added 500 ppm methoxyethanol to conditioned MgTFSI_2_/G2 solutions. Figure 7B shows that the resultant CE decreases from 88% to ∼57%, and the plating/stripping current density decreases significantly. Both are restored upon performing a subsequent conditioning process. These results further exemplify that the organic impurities in addition to water in the glyme solvent inhibit the performance and suggest the necessity of conditioning, which can aid to remove these species.

Additionally, in addition to impurities in the as-received solvents, process-related detrimental substances may form in the prepared electrolyte, which could be treated as impurities as well. A previous study from Barile et al. (2014) identified the presence of high molecular weight species in the freshly synthesized MACC electrolyte. These oligomers formed due to ring-opening polymerization of THF during electrolyte formulation can impede the performance, but they were removed by the conditioning process to improve the performance. Unlike in MACC, the ESI-MS data in Supplementary Figure S4 confirm that no oligomeric species is present in the as-prepared MgTFSI_2_/G2 (distilled) solution, indicating no such ring-open polymerization for an acyclic ether solvent. Table 2 summarizes the major cation speciation as identified from the ESI-MS for both freshly prepared and conditioned solutions. Both spectra show Mg (G2)_2_^2+^ and Mg (TFSI) (G2)^+^ species as the two most intense peaks. However, their relative ratio increases from 0.6:1 in the fresh electrolyte to ∼4.9:1 in the conditioned electrolyte. This suggests that after conditioning, most Mg^2+^ are solvated by two G2 molecules only without forming contact Mg–TFSI ion pairs, or some TFSI anions are released from the Mg (TFSI) (G2)^+^ structure during cycling. This preferred structure can facilitate fast ion exchange, which might be responsible for the highly reversible magnesium plating and a better Coulombic efficiency in the conditioned solution. On the other hand, four new species are recognized only from the conditioned electrolyte (see Table 2 for the possible structure), although their concentrations are much lower. They contain nitrogen and fluorine that are liberated from TFSI^−^, indicating the decomposition of the salt itself. This observation is consistent with the results revealed by our XPS and EDS data on the black mass deposited onto the Mg electrode in Supplementary Figure S5 and the other literature (Yoo et al., 2017). TFSI anions or [Mg (TFSI)]^+^ ion pairs could be easily attacked by the free nucleophilic OH^−^ from water/alcohol impurities or by simply releasing from the Mg (OH)_2_ surface, resulting in bond breaking of C–S of TFSI (Yu et al., 2017). It is likely that gains in CE by conditioning/continuous cycling may come at the expense of the decomposition of MgTFSI_2_, particularly with the presence of impurities. Thus, the instability of MgTFSI_2_ itself at the Mg anode/electrolyte interface is another important factor that limits the electrochemical performance (to reach CE > 95%). Other relatively stable salts such as magnesium tetra (hexafluoroisopropyl)borate (Mg [B (hfip)_4_]_2_ and magnesium tetrakis (perfluoro-tert-butoxy)aluminate (Mg [TPFA]_2_), or co-salt magnesium triflate [Mg (OTf)_2_] with MgCl_2_ in glymes, are now being developed to overcome this limitation (Deivanayagam et al., 2019; Lau et al., 2019; Dlugatch et al., 2021; Ren et al., 2021).

**TABLE 2:** Mass spectra speciation of the major cation species probed by ESI-MS for freshly prepared and conditioned 0.5 M Mg (TFSI)_2_/G2 solutions.

Solution	m/z	Compound/empirical formula	Ratio (%)
Fresh	142	Mg (G2)_2_^2+^	37.2
	438	Mg (TFSI) (G2)^+^	62.8
Conditioned	142	Mg (G2)_2_^2+^	80.6
	184	Mg (G2)_2_(CF_3_)^2+^	1.6
	220	Mg (G2)_2_(C_6_H_14_O_3_N)^2+^	0.3
	438	Mg (TFSI) (G2)^+^	16.3
	618	Mg (TFSI) (G2)_2_(CH_3_ON)^+^	1.0
	947	Mg_3_ (TFSI)_2_ (G2)_2_F_3_^+^	0.2

The purity of the salt also affects the Mg plating/stripping efficiency. Mg (TFSI)_2_ salt is highly hygroscopic and forms a crystalline hexahydrate with residue water, making it extremely hard to eliminate the water completely from the salt (Ma et al., 2017). It is difficult to determine the water content in the salt and efficacy of electrochemical conditioning to remove it from salt. Drying salts at an elevated temperature (140–180°C) appears to be insufficient, and other synthesis/purification techniques, such as recrystallization of commercial salts, should be considered in the future.

As such, we propose that electrochemical conditioning is necessary for the MgTFSI_2_/glyme electrolytes prior to battery operation. This charge (Q)-controlled electrolytic conditioning protocol we developed to remove impurities is controllable, quantitative, and repeatable from batch to batch. Also, this process is practical, requires no special synthesis/purification apparatus in the glovebox, and provides a safe method to implement in the lab that does not make use of highly reactive materials such as Na and K. We suggest this protocol as a solution to many users to accurately assess the electrolyte performance based on the MgTFSI_2_/Gx system.

### 2.5 Aging and storage

**FIGURE 8:**
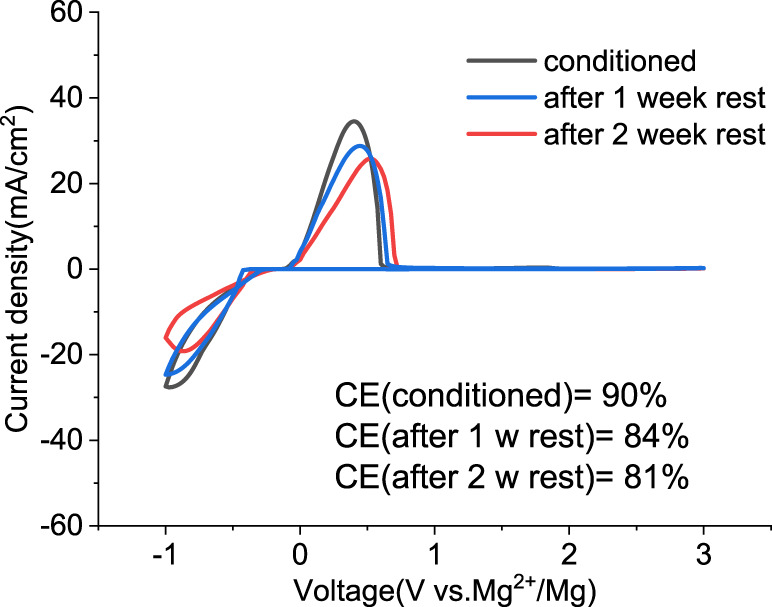
Cyclic voltammograms of the conditioned MgTFSI_2_/MgCl_2_ in dried G2 and after resting for 1 week and 2 weeks at OCP.

We investigated the stability of the conditioned electrolyte under resting conditions in a sealed cell stored in an inert atmosphere for extended times. Figure 8 compares the CV of the solution containing MgCl_2_/MgTFSI_2_ in dried G2 after 1 or 2 weeks at OCP. The overpotential after 1 week resting remains the same as the conditioned but increases slightly after 2 weeks of resting. The CE decreases by ∼5% after every week of storage. The results indicate that the aging of MgCl_2_/MgTFSI_2_ in G2 is not as severe as the MACC in THF, which undergoes ongoing THF ring-opening polymerization (Barile et al., 2014); however, we observed that this stability is dependent on the storage environment. For example, the glovebox atmosphere should be maintained at < 1 ppm O_2_ and H_2_O.

## 3 Conclusion

This study presents the identification of impurity in glyme solvents, which have been extensively used in Mg batteries. GC-MS shows that in addition to water, other protic organic contaminants (R-OH) like butanol affect the reversibility of Mg cycling significantly. Purifying methods to remove impurities including molecule sieve drying, Na/K distillation, additive addition, and hybrid salt were evaluated and compared. We conclude that the removal of impurity is vital to achieving reversible Mg electrochemistry. We proposed a facile conditioning protocol to further improve the performance of MgTFSI_2_/Gx electrolytes by galvanostatic cycling at different currents. The conditioned electrolyte after Q-controlled electrochemical conditioning demonstrates highly reversible Mg deposition and a large current density, characteristics that are necessary for practical Mg-ion batteries. GC-MS data reveal that residual water during the electrolyte preparation and some organic impurities can be effectively eliminated by a conditioning process. ESI-MS data show that the speciation favoring Mg complexes such as Mg (G2)_2_^2+^ enables much more reversible Mg deposition from the conditioned solution. New decomposition species are also formed during cycling because of the instability of MgTFSI_2_ itself. This work also illustrates that glyme solvents may undergo subtle aging processes during storage.

## 4 Experimental and methods

### 4.1 Chemicals

All chemicals were purchased from Sigma Aldrich, unless otherwise stated. Monoethylene glycol dimethyl ether (G1, 99.5%), diethylene glycol dimethyl ether (G2, 99.5%), and triethylene glycol dimethyl ether (G3, 99%) were received and stored in an argon-filled glovebox. The solvents used as-is, that is, without any treatment, for electrolyte formulations were called “as-received” in the following text. Solvents were dried prior to use over molecular sieve (alumina 3 Å) for 48 h in the glovebox in a stationary mode and named “dried by MS” in the text. For comparison, all the solvents were purified by vacuum distillation (25 cm Vigreux column) over liquid Na/K alloy; in the main text, they are referred to as “distilled solvent.” Although this method provides solvents in a purified, dry form, the use of such highly reactive agents can be quite dangerous for both the novice and the experienced researcher. Extreme caution and PPE (hood, blast shields, face shield, and protective and fire-resistant clothing) should always be used. Considering the danger and laborious work introduced by using metallic Na/K, purification by calcium hydride was also tried in this work to evaluate its feasibility in practice; the solvent was referred to as “dried by CaH_2_.” Typically, fresh ground CaH_2_ (∼10 g) was added to 1 L of solvent in a side-arm Schlenk flask, and the solvent was allowed to stir over CaH_2_ overnight. Once finished, all the solvents were stored in an inert atmosphere (i.e., glovebox). The water content in these samples was determined by Karl Fischer titration (Mettler-Toledo).

Mg (TFSI)_2_ (99.5%, solvionic) and MgCl_2_ (99.9%) were vacuum-dried at 150°C for 24 h before use. The electrolytes contained different mixtures were stirred at room temperature for 1 day to make uniform solutions. All electrolytes were prepared in a wet-chemical glovebox and then were transferred into another glovebox, which was equipped with electrochemistry testing apparatus using a sealed mason jar without exposure to air.

### 4.2 Gas chromatography—mass spectrometry measurement

The impurities in the solvents were identified using the Clarus 600/560D gas chromatograph with a mass spectrometer detector. An autosampler (Perkin Elmer) was used to make the sample injections. The inlet was equipped with a split/splitless injector and a nonpolar Supelco SLB™-5 ms (30 m × 0.25 mm I.D. × 0.25 µm) film capillary column. A constant helium flow rate of 1.0 ml/min was used as the carrier gas. An inlet temperature was set at 220°C, and the oven temperature was programmed to heat from 50 to 250°C at 20°C/min. The final oven temperature was held for 5 min to allow all the compounds to elute. The overall measurement time was 15 min. The mass ranges from 15 to 300 m/z, and the event time was 0.1 s in a scan mode. The mass spectrometer was run in the electron impact ionization (EI) mode. The temperature of the ion source was set to 180 C, and the filament was operated at a voltage of 70 V. Data were processed by TurboMass™ software with the commercial searchable libraries (NIST) for automatic peak detection and spectral deconvolution.

### 4.3 Electrochemistry

**FIGURE 9:**
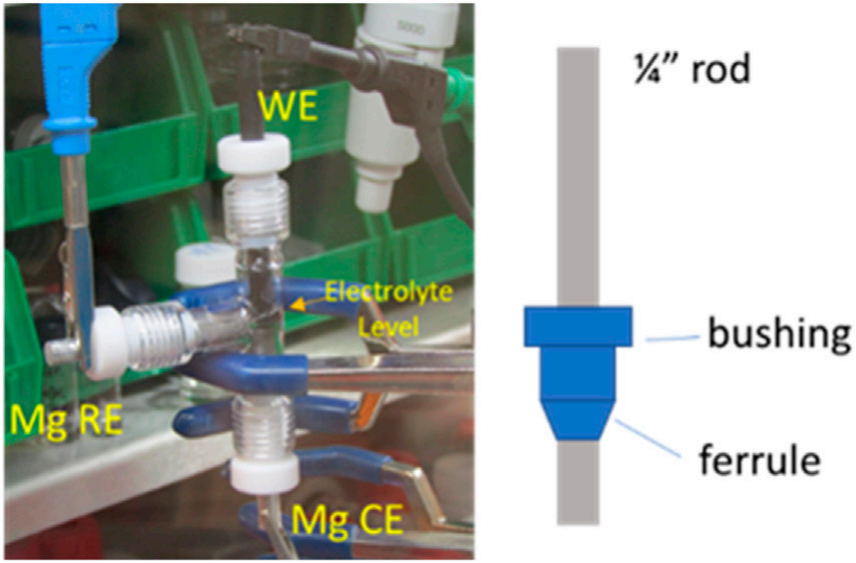
Photograph of an assembled three-electrode cell in the glovebox. It is mounted in a standard three-prong lab clamp.

Cyclic voltammograms of each electrolyte were carried out using a customized three-electrode glass T-cell (ACE Glass), as shown in Figure 9. The bushing and ferrule were made of polytetrafluoroethylene (PTFE) to resist organic solvents. All glassware and sealing parts were dried in an oven at 120°C overnight before transferring into the glovebox. A Pt disk was cleaned by immersing it in 1 M HNO_3_ before testing and was used as the working electrode (2 mm in diameter, CH instruments, Austin, TX). Mg rods, polished in the glovebox to expose fresh metal surface, were used as the counter and reference electrodes (99.9% purity). To ensure the consistency and comparison of the electrochemistry activity between different electrolytes, 1.5 ml of solution was introduced in the cell to keep the same electrolyte level. The distance between each electrode was fixed for every experiment. Cyclic voltammetry (CV) was performed using a Gamry electrochemical workstation in the glovebox.

### 4.4 Liquid chromatography–electrospray ionization–mass spectrometry (LC-ESI-MS)

Mass spectrometry measurements were taken using an Agilent Technologies 1260 Infinity liquid chromatograph equipped with an Agilent 6120 Quadrupole ESI mass spectrometer. A direct infusion method with sample *via* airtight Hamilton syringes was used to minimize exposure to an ambient atmosphere. The detector capillary voltage was 3000 V, the drying gas (N2) flow rate was 12 L/min, the nebulizer pressure was 35 psig, the drying gas temperature was 350°C, and the fragmentor voltage was 70 V. The spectrometer measured a range of m/z values from 150 to 2,000.

### 4.5 X-ray photoelectron spectroscopy

A PHI VersaProbe II was used for XPS measurements of Mg electrodes after cycling. This system was attached to an Ar-atmosphere glovebox, and the samples were transferred to the XPS through a glovebox without exposure to air. A 100-W X-ray beam was focused to a 100 µm diameter. A high-resolution spectrum for each individual element was taken with a 23.5 eV pass energy over 30 scans. Spectra were calibrated against a C–C 284.8 eV carbon peak.

## Data Availability

The original contributions presented in the study are included in the article/Supplementary Material; further inquiries can be directed to the corresponding authors.
