# Supplementary material for: Toward practical issues: Identification and mitigation of the impurity effect in glyme solvents on the reversibility of Mg plating/stripping in Mg batteries
Source: Front Chem. 2022 Aug 12;10:966332. doi: 10.3389/fchem.2022.966332 (PMC9413053; doi:10.3389/fchem.2022.966332)
Supplement: Supplementary file 1 [file DataSheet1.docx]

**Supporting information**

**Towards practical issues: Identification and mitigation of the impurity effect in glyme solvents on the reversibility of Mg plating/stripping in Mg battery**

**Zhenzhen Yang,^1,2,*^ Mengxi Yang,^2^ Nathan T. Hahn,^2,3^, Justin Cornell,^2,4,^ Ira Bloom,^1^ Chen Liao,^1,2^ Brian Ingram,^1,2^ Lynn Trahey ^2*^**

1: Chemical Sciences and Engineering Division, Argonne National Laboratory, Lemont, Illinois 60439, United States

2: Joint Center for Energy Storage Research, Argonne National Laboratory, Lemont, Illinois 60439, United States

3: Material, Physical and Chemical Sciences Center, Sandia National Laboratories, Albuquerque, New Mexico 87185, United States

4: Material Science Division, Argonne National Laboratory, Lemont, Illinois 60439, United States

**Corresponding authors:** [yangzhzh@anl.gov](mailto:yangzhzh@anl.gov), [Trahey@anl.gov](mailto:Trahey@anl.gov)

**Figure S1.** Common materials and routes for glyme synthesis adapted from the literature(Tang and Zhao, 2014).

**Figure S2**. Cyclic voltammogram (CV) for the electrolyte with 0.5M MgTFSI_2_ in G1 dried by CaH_2_ method. Pt was used as the working electrode and a scan rate of 25 mV s^-1^. Water content was 19 ppm after CaH_2_ treatment.


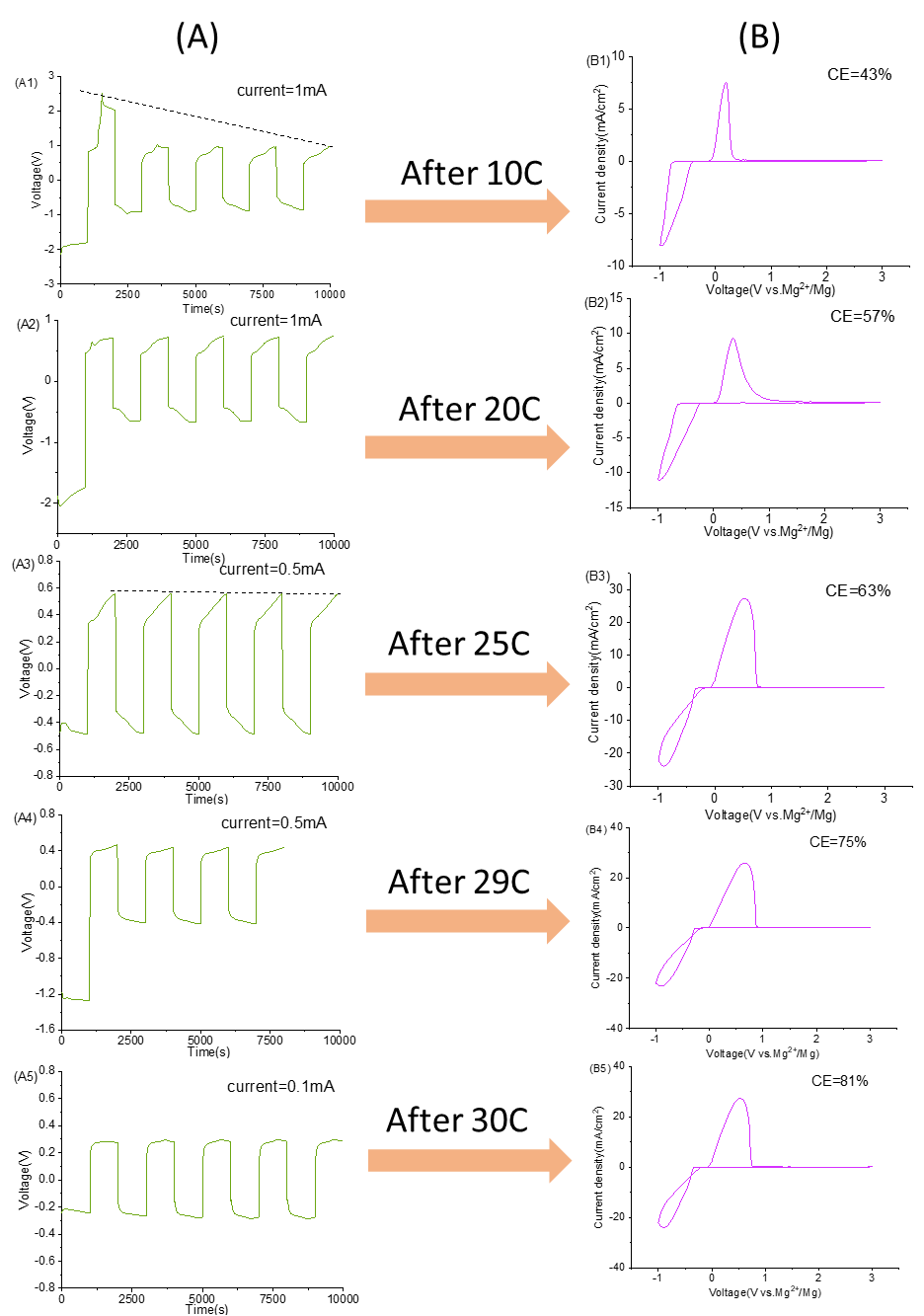


**Figure S3.** Detailed conditioning process using solution in as received G2 (0.25M MgTFIS_2_ + 0.25M MgCl_2_) as an example. (A) Voltage vs. Time profiles to show evolution of electrochemical response with varying conditioning current. (B) CV measurement after each conditioning process to evaluate if reasonable good columbic efficiency achieves. Scan rate: 25mV/s. The charged shown here was the accumulated charge after every step used for conditioning.


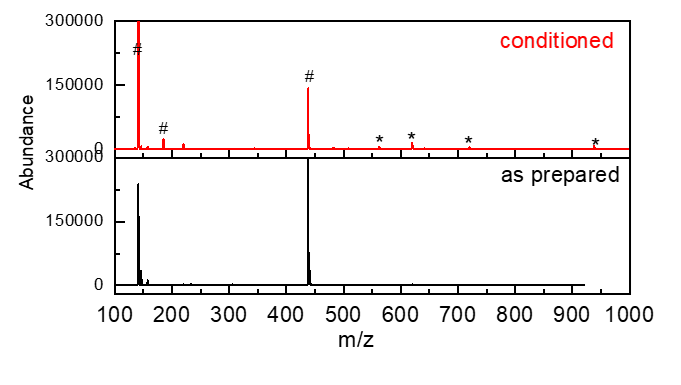


**Figure S4.** ESI-MS of the freshly prepared and conditioned 0.5M MgTFSI_2_/G2 (distilled) solutions. Peaks marked with an “#” represent the complexes existing in both solutions and peaks marked with an “*” represent the species only present in conditioned electrolyte.


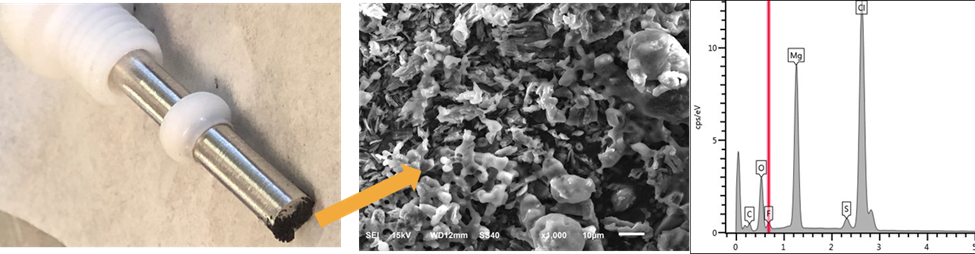


**Figure S5.** (A) Optical image of the black deposit on Mg rod from “as received” solution after conditioning cycles. During long-term cycling or conditioning process, we found black mass precipitated on the Mg metal rod over time for all the solutions we studied in this work. (B) SEM image of the black deposit. (C) EDS of the decomposition product. Compounds containing Mg, C, F, S, O and Cl were detected from EDS analysis, evidence from the salt decomposition of MgTFSI_2._

**References:**

Tang, S. & Zhao, H. (2014). Glymes as versatile solvents for chemical reactions and processes: from the laboratory to industry. *RSC Adv.,* 4**,** 11251-11287.
